# Impact of Sampling Period on Population Pharmacokinetic Analysis of Antibiotics: Why do You Take Blood Samples Following the Fourth Dose?

**DOI:** 10.3390/ph13090249

**Published:** 2020-09-16

**Authors:** So Won Kim, Dong Jin Kim, Dae Young Zang, Dong-Hwan Lee

**Affiliations:** 1 Department of Pharmacology, Asan Medical Center, University of Ulsan College of Medicine, Seoul 05505, Korea; kswlab2015@gmail.com; 2 Drug Evaluation Department, National Institute of Food and Drug Safety Evaluation, Ministry of Food and Drug Safety, Osong, Cheongju 28159, Korea; nobaes79@gmail.com; 3 Division of Hematology-Oncology, Department of Internal Medicine, Hallym University Sacred Heart Hospital, Hallym University College of Medicine, Anyang 14066, Korea; fhdzang@gmail.com; 4 Department of Clinical Pharmacology, Hallym University Sacred Heart Hospital, Anyang 14066, Korea

**Keywords:** population pharmacokinetics, antibiotics, sampling period, first dose, fourth dose

## Abstract

To date, many population pharmacokinetic models of antibiotics have been developed using blood sampling data after the fourth or fifth dose, which represents steady-state levels. However, if a model developed using blood sampled after the first dose is equivalent to that using blood sampled after the fourth dose, it would be advantageous to utilize the former. The aim of this study was to investigate the effect of blood sampling after the first and/or fourth drug administration on the accuracy and precision of parameter estimates. A previously reported robust, two-compartment model of vancomycin was used for simulation to evaluate the performances of the parameter estimates. The parameter estimation performances were assessed using relative bias and relative root mean square error. Performance was investigated in 72 scenarios consisting of a combination of two blood sampling periods (the first and fourth dose), two total clearances, three infusion times, and four sample sizes. The population pharmacokinetic models from data collected at the first dose and those collected at the fourth dose produced parameter estimates that were similar in accuracy and precision. This study will contribute to increasing the efficiency and simplicity of antibiotic pharmacokinetic studies.

## 1. Introduction

The era of precision medicine is approaching, which both prevents and treats diseases by considering the genetic makeup, environment, and lifestyle of each person [1]. Unlike the one-size-fits-all approach, which considers the average patient, precision medicine can significantly improve patient safety and clinical outcomes by comprehensively reflecting an individual patient’s status comprehensively [2,3,4]. To realize precision medicine in real-world clinical practice, a variety of obstacles should be overcome, including not only genotypes but also co-administered drugs, food, pathophysiologic changes, and the use of medical devices such as hemodialysis or extracorporeal membrane oxygenation (ECMO) machines. Model-informed precision dosing (MIPD) is a method that can overcome the practical difficulties of realizing precision medicine. The goal of MIPD is to improve the treatment efficacy and reduce the side effects for each patient through personalized treatment using a modeling approach. Examples of these approaches include population pharmacokinetics/pharmacodynamics (Pop PK/PD), physiologically based PK, and quantitative systems pharmacology [5,6].

Robust Pop PK/PD models are essential for precision pharmacotherapy. Pop PK/PD modeling and simulation is a tool to analyze the dose–exposure–response relationship quantitatively. This provides population parameter estimates using a nonlinear mixed-effects model and individual parameter estimates, concentrations, and responses by identifying sources of variability, such as age, gender, body weight, renal function, and genotype [7]. The population approach has become an essential tool in drug development because it can save time and money by integrating knowledge to aid in determining the study design and dosage regimens or making go/no-go decisions in drug development [3]. Pop PK/PD also plays a role in solving unmet needs in medical practice. Since clinical trials do not reflect all real-world scenarios, there is inevitably a gap between clinical trials and clinical practice [8,9]. Pop PK/PD studies after drug approval bridge this gap, allowing personalized medicine for patients including subpopulations not included in the clinical trials. In addition, it is possible to perform real-time precision medicine by using Bayesian models that reflect various factors such as genotype, demographics, and pathophysiology [10,11,12].

Pop PK/PD analysis of antibiotics overcomes the antibiotic resistance and poor efficacy of suboptimal doses or toxicity of high doses because it optimizes the dosage regimen using a Pop PK model and Monte Carlo simulation (MCS). If the concentration range causing antibiotic resistance is known, a Pop PK model can determine the dosing regimen and adjust the patient’s drug concentration to be within the concentration range that does not cause resistance [13]. Therefore, a validated Pop PK model is indispensable for adjusting the dosage regimens of antimicrobials and predicting treatment outcome with MCS [13,14,15]. To develop a useful Pop PK model, the following blood sampling-related factors are considered: number of subjects, number of samples per subject, nominal sampling times, and blood sampling after single or multiple doses [16,17]. In most Pop PK studies of antibiotics, blood sampling routinely occurs from the fourth to fifth drug administration period to ensure that steady state levels have been reached [18,19,20,21,22,23]. However, such a sampling scheme may have the following disadvantages.

- Recording all doses and times accurately until steady state is achieved can be cumbersome. If there is an error in the record, model development is adversely affected.
- It is difficult to estimate PK parameters accurately and find a significant covariate that affects PK when a patient’s pathophysiological state continues to change until the drug’s steady state is reached.
- The enrolled patient is likely to drop out before steady state is achieved.

Thus, development of a model that uses blood sampling after the first dose, which is equivalent to a model that use sampling after the fourth dose, would be advantageous in terms of time and cost to conduct clinical research. The purpose of this study was to investigate the effect of blood sampling after the first and/or fourth drug administration on the accuracy and precision of PK model parameter estimates.

## 2. Results

**Figure 1:**
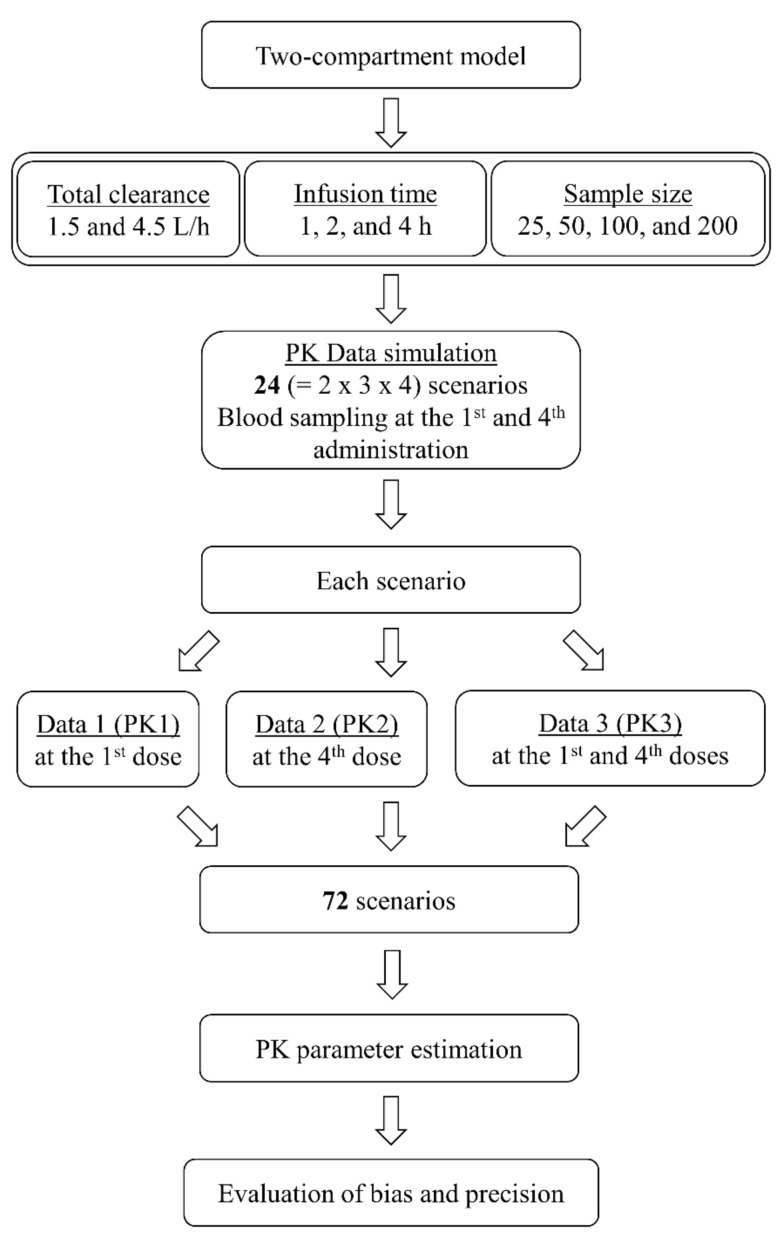
Flowchart for simulation and estimation.

The accuracy and precision of parameter estimates (θ, ω^2^, θ_ε_) for a total of 72 scenarios were evaluated using relative bias (rBias) and relative RMSE (rRMSE), respectively (Figure 1). Because the accuracy and precision for the 2-h infusion data were similar to those for the 1-h infusion data, twenty-four scenarios for the 2-h infusion data were not included in the results. In general, the parameter estimates of the 1-h infusion data were more accurate and precise than those of the 4-h infusion data. Comparing all the parameter estimates for total clearance (CL) values of 1.5 and 4.5 L/h, there was little difference in rBias and rRMSE. In most cases, both the PK1 and PK2 datasets yielded unbiased and precise parameter estimates and the PK3 dataset yielded the most accurate and precise estimates.

**Figure 2:**
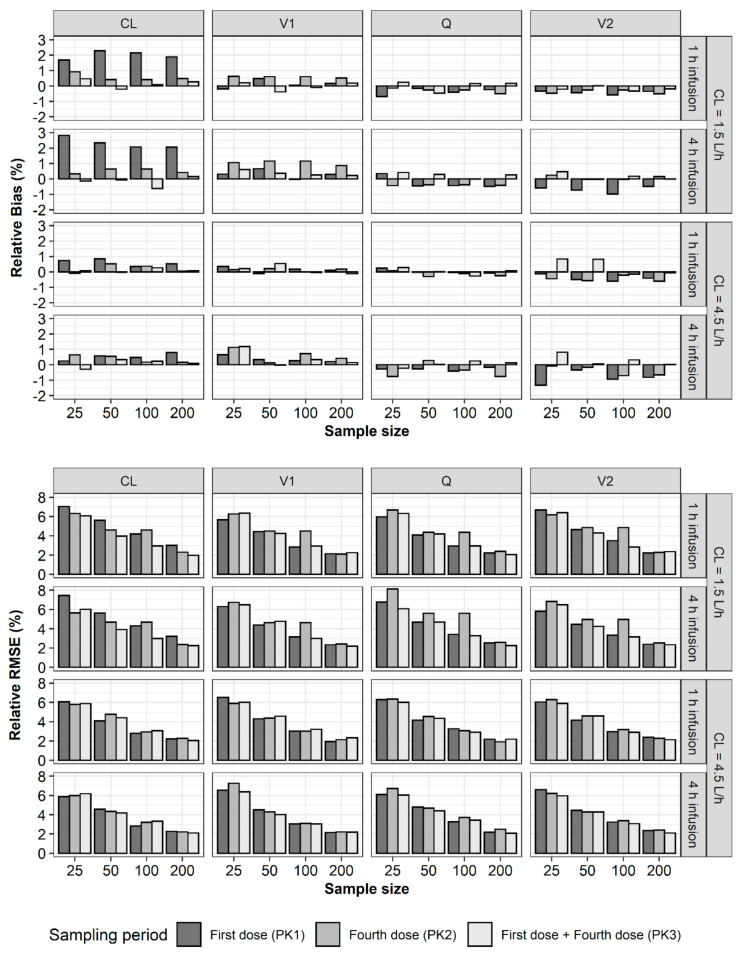
Relative bias (**upper**) and relative RMSE (**lower**) of structural pharmacokinetic parameters estimates for two-compartment model of vancomycin (CL—total clearance; V1—volume of the distribution of the central compartment; V2—volume of distribution for the peripheral compartment; Q—intercompartmental clearance between V1 and V2).

**Figure 3:**
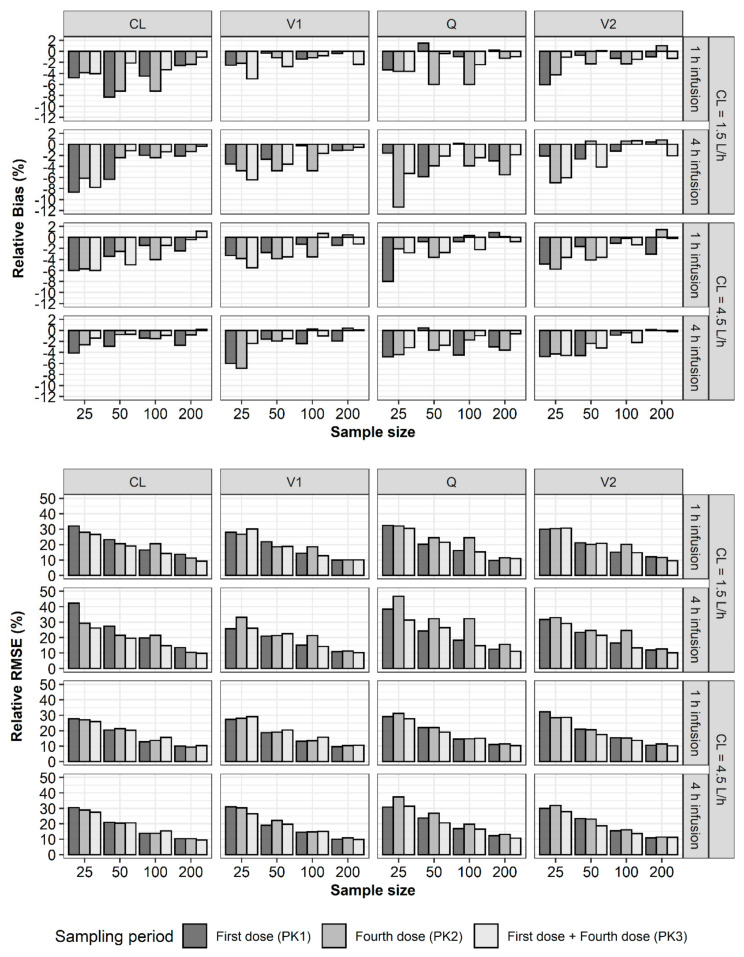
Relative bias (**upper**) and relative RMSE (**lower**) of between-subject variability of structural pharmacokinetic parameters for two-compartment model of vancomycin (CL—total clearance; V1—volume of the distribution of the central compartment; V2—volume of distribution for the peripheral compartment; Q—intercompartmental clearance between V1 and V2).

**Figure 4:**
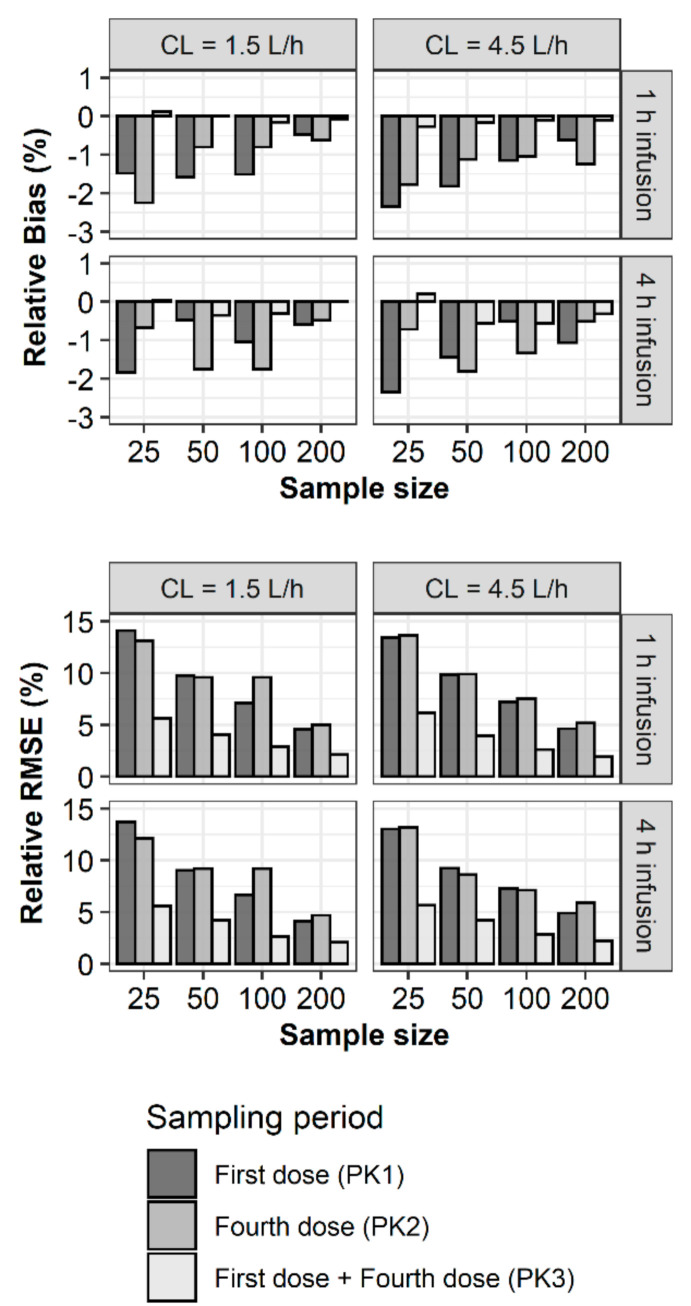
Relative bias (**upper**) and relative RMSE (**lower**) of residual variability in vancomycin concentrations.

For the structural parameters of the two-compartment model, rBias and rRMSE of all the parameter estimates were less than 15% and 35%, respectively (Figure 2). In the case of rBias, no value exceeded 3%. When CL with a true value of 1.5 L/h was estimated using PK1, PK2, and PK3, rBias was the largest with PK1. If the CL was 1.5 L/h, the rBias of the central volume of distribution (V1) was the largest with PK2. In other scenarios, the rBias values of the estimates were less than 1.5% and showed no noticeable trends or differences between PK1, PK2, and PK3. In the case of rRMSE, no value exceeded 8%. The rRMSE showed similar values and tended to decrease with increasing sample size. For the between-subject variability (BSV) in the structural parameters, the rBias values of all the parameter estimates were less than 15%, while some of the rRMSE values exceeded 35% (Figure 3). When estimating with PK1, if the sample size was 25, the rRMSE of CL and intercompartmental clearance (Q) exceeded the precision criteria. When estimating with PK2, if the sample size was 25, the rRMSE of Q exceeded 35%. In other scenarios, the rRMSE showed no noticeable trends or differences among PK1, PK2, and PK3 when the sample sizes were identical. The rBias and rRMSE values tended to decrease with increasing sample size. For the residual variability (RV) of the PK profiles, the rBias and rRMSE values were less than 15% and 35%, respectively. The rBias and rRMSE values were considerably smaller when estimated using PK3 and tended to decrease as the sample size increased (Figure 4).

## 3. Discussion

**Figure 5:**
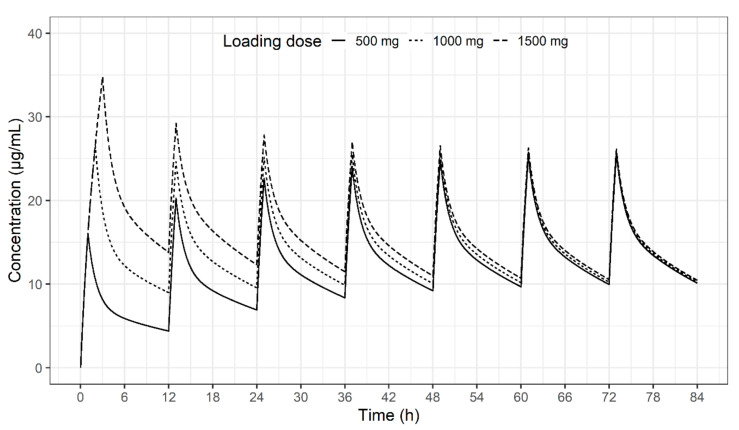
Concentration-time profile of vancomycin following three different loading doses and a maintenance dose of 500 mg every 12 h at a dose of 500 mg.

Pop PK/PD analysis is a powerful tool for implementing personalized medicine, and this power is derived from the quality and quantity of the data resulting from the study design. From a PK perspective, the reason for steady-state blood sampling in PK model development was questionable. The use of blood sampling at the fourth or fifth dose in antibiotic PK studies originated from clinical practice in therapeutic drug monitoring (TDM). When TDM is performed, the peak and trough concentrations are measured at steady state to confirm that the current dosage regimen is appropriate for inclusion in the therapeutic range. It is generally assumed that a steady state has been reached after 4–5 times the half-life, which is considered to have been achieved usually after administration of 4–5 doses. Even in cases where the loading dose does not reach or exceed the expected therapeutic range, a steady state is reached by the maintenance dose after 4–5 times the half-life (Figure 5). However, this assumption is not correct when renal function decreases and the half-life is prolonged. In this case, even if the medication was given 4–5 times, only 2–3 times the half-life may have passed [24]. Another problem with steady-state sampling is that any changes occurring before steady state attainment will hinder the creation of a useful model. If it is possible to construct a model using blood sampling data acquired after the first drug dose, the ease of conducting Pop PK studies will facilitate the study of precision antibiotic dosing. In the present study, we explored the effects of blood sampling period on the development of PK models of vancomycin. We hypothesized that blood sampling does not need to be performed at steady-state levels to build a useful Pop PK model of antimicrobials but may be performed after the first drug administration.

Vancomycin PK profiles were described by a one-compartment model in a sparse sampling scheme [25], while they were explained by a two-compartment model in a dense sampling scheme [26,27,28]. We chose a two-compartment model for this study because ability to predict the concentration of the two-compartment model is superior to that of the one-compartment model [29,30,31]. Another reason is that a large bias in the area under the curve (AUC) of PK profiles can occur by ignoring a steep distribution phase slope. If a one-compartment model is used to predict AUC for a patient with a two-compartment PK property, it might result in administering inappropriate doses [17]. A revised consensus guideline for the therapeutic monitoring of vancomycin suggests the PK/PD target as an AUC/MIC ratio of 400–600 to increase efficacy for MRSA infections with a MIC ≤ 1 mg/L and decrease the likelihood of acute kidney injury. It also recommends Bayesian-guided AUC monitoring [32].

In our study, the overall rBias and rRMSE were considerably smaller than those in previous simulation studies [33,34,35]. This is due to our use of a robust model for simulation and linearity [28]. In previous simulation studies, the nonlinear PK models showed relatively large rBias and rRMSE values compared to those in our linear PK model. For the blood sampling period, both the single-dose data (PK1) and multiple-dose data (PK2) were appropriate for developing a population PK model. PK3, which combined the PK1 and PK2 data, usually showed the best accuracy and precision because there was twice the amount of data with the same sample size. Several relatively large rBias and rRMSE values decreased with increasing sample size. For PK1, the rBias of the CL estimates was large when the CL value was as small as 1.5 L/h because the sampling time was too short to estimate CL accurately, while the half-life was long. In this case, the overprediction of CL appeared due to the lack of blood samples at the elimination phase, resulting in a steep slope for this part. For BSV and RV, rBias was usually calculated as a negative number. This phenomenon is called shrinkage, which indicates that the distribution of individual parameters or observations obtained from mixed-effects models is narrower than that assumed to estimate the random variables for parameters or observations [36,37]. In the present study, the size of negative bias for RV was smaller than the BSV estimated using variance because RV was estimated using standard deviation. Shrinkage has also been seen in previous simulation studies and appears when the sample size is small, sampling times are inappropriate, or number of observations per subject is insufficient to estimate the real distribution [33,34,35]. Shrinkage of 20–30% or more may weaken, falsely strengthen, or distort the correlation between parameters or between parameters and covariates [36].

Small differences can have a large effect, and vice versa. If small pathophysiological changes accumulate, noise in the clinical data may increase, and a model with similar predictive ability could be derived despite a large difference in study design with respect to the blood sampling period. If these two phenomena occur simultaneously, it is much better to develop a good model for blood sampling that uses the first drug dose. Of course, it is best for models to reflect various pathophysiology, but the first dosing period is better if one must choose between the first and the fourth dose. We conducted this study to promote research on antibiotic therapy to fulfill an unmet medical need for precision dosing in the era of precision medicine, which has not been realized by examining only genotypes. To implement precision medicine in clinical practice, it should be possible to reflect not only pharmacogenomics but also PK/PD changes in real-world practice. For patients using warfarin, the genotypes of cytochrome P450 (CYP) 2C9 and vitamin K epoxide reductase complex subunit 1 should be considered [38]. The risk of rhabdomyolysis in patients using statins increases with the concurrent use of CYP3A4 inhibitors such as antifungal agents (itraconazole, ketoconazole, and fluconazole) or macrolide antibiotics (erythromycin and clarithromycin) [39]. Grapefruit juice, which inhibits intestinal CYP3A4, significantly increases the blood concentration of simvastatin [40]. As renal function decreases or increases with pathophysiological alterations in critically ill patients, target concentrations of antimicrobial agents become difficult to achieve [41,42]. Precise dosing for patients undergoing dialysis is difficult due to the interaction of dialysis methods with various physicochemical properties such as size, distribution volume, protein binding, and water solubility of drugs [43]. Drug concentrations in patients receiving ECMO may be lowered due to increased volume of distribution and decreased clearance of the drug [44].

This study has some limitations. First, the results of this study were derived by simulation. It is expected that more noise will be generated when a clinical study is conducted in real-world practice, although actual differences can be investigated. Second, a model without covariates was used in this simulation. Although a model with covariates would be more realistic, we thought that clearly distinguishing only the difference in the blood sampling period in such a model would be difficult. Third, this study assumed PK linearity and did not consider nonlinearity. However, most drugs are used within the linearity range of PK. In addition, in some drugs with nonlinear PK, concentrations that exhibit nonlinearity may also appear at the first dose as well as at the fourth dose. In this case, a model should be constructed applying a nonlinear PK model such as the Michaelis–Menten equation, and internal evaluation with external validation with additional data as well as goodness of fit plots and visual predictive checks should be performed. Fourth, we neglected that the patients’ pathophysiology and their PK parameters can change with time and treatment. To find these changes and reflect them in the model, data at the first dose are not sufficient. We think that dense sampling data at the first dose and sparse data at the steady state might be required to address the changes.

In conclusion, the Pop PK models from data collected after the first dose and data collected after the fourth dose were similar in the accuracy and precision of parameter estimates, although it was best to draw blood samples after both the first dose and attainment of steady-state levels. Considering the difficulty of conducting an actual clinical study, drawing blood samples after the first drug dose seems adequate. The results of this study can help in the design and implementation of future Pop PK studies of antibiotics.

## 4. Materials and Methods

### 4.1. Pharmacokinetic Simulation and Estimation

A previously reported robust two-compartment model of vancomycin was used for PK data simulation [28]. The model was developed to describe the vancomycin PK profiles in adult patients undergoing veno-venous or veno-arterial ECMO. The structural PK parameter values were 24.2 L, 32.3 L, and 11.2 L/h for V1, the volume of distribution for the peripheral compartment (V2), and Q, respectively. CL values of 1.5 and 4.5 L/h were evaluated to explore the effect of CL on the parameter estimation performance, while the reported CL was 2.83 L/h.

The concentration–time profiles of vancomycin after the first and fourth doses were generated using a dose of 1000 mg every 12 h, three infusion times (1, 2, and 4 h), and four population sample sizes (25, 50, 100, and 200). The blood sampling times were before (0 h) and 1, 2, 4, and 12 h after the start of the first and fourth 1-h infusions; 0, 2, 3, 5, and 12 h after the start of the first and fourth 2-h infusions; and 0, 4, 5, 9, and 12 h after the start of the first and fourth 4-h infusions. Twenty-four PK scenarios were constructed with three datasets as follows: concentrations at the first dose (PK1), at the fourth dose (PK2), or at both the first and fourth doses (PK3) (Figure 1).

For the two-compartment linear PK model, the equation for the change in concentration over time from the start to completion of the intravenous infusion becomes:

$$C_{P}=R_{inf}\times \left\{\frac{A}{\alpha }\times \left(1-e^{\left(-\alpha *t\right)}\right)+\frac{B}{\beta }\times \left(1-e^{\left(-\beta *t\right)}\right)\right\}$$

where *R_inf_* is infusion rate, *t* is time, $\alpha =\frac{\left(k_{10}+k_{12}+k_{21}\right)+\sqrt{{\left(k_{10}+k_{12}+k_{21}\right)}^{2}-4\left(k_{10}\times k_{21}\right)}}{2}$, $\beta =\frac{\left(k_{10}+k_{12}+k_{21}\right)-\sqrt{{\left(k_{10}+k_{12}+k_{21}\right)}^{2}-4\left(k_{10}\times k_{21}\right)}}{2}$, $A=\;\frac{\alpha -\;k_{21}}{V1\times \left(\alpha -\beta \right)}$, and $B=\;\frac{k_{21}-\beta }{V1\times \left(\alpha -\beta \right)}$. The micro-rate constants were derived using: $k_{10}=\;CL/V1$, $k_{12}=\;Q2/V1$, and $k_{21}=\;Q2/V2$.

Then, changes in the concentration over time after completion of the intravenous infusion were calculated by

$$C_{P}=R_{inf}\;\times \left\{\frac{A}{\alpha }\times \left(1-e^{\left(-\alpha *t_{inf}\right)}\right)\times e^{\left(-\alpha *t\right)}+\frac{B}{\beta }\times \left(1-e^{\left(-\beta *t_{inf}\right)}\right)\times e^{\left(-\beta *t\right)}\right\}$$

where *t_inf_* is the infusion time.

The coefficient of variation of random effects for BSV and RV were set at 30% for the four structural PK parameters and 10% for concentrations, respectively. An exponential error model for BSV and a proportional error model for RV were applied for the simulation and estimation as follows:

$$\theta_{i}=\;\theta \times exp\left(\eta_{i}\right)$$

$$y_{ij}=\;\hat{y_{ij}}\;\times \left(1+\;\theta_{\varepsilon }\;\times \;\varepsilon_{ij}\right)$$

where $\theta_{i}$ is the *i*th individual PK parameter, θ is the population typical value, is a normally distributed random variable with mean zero and variance of ω^2^, $y_{ij}$ is the observed concentration for the *i*th individual at time *j*, $\hat{y_{ij}}$ is the predicted concentration for the *i*th individual at time *j*, $\varepsilon_{ij}$ is a normally distributed random variable with mean zero and variance of 1, and $\theta_{\varepsilon }$ is the coefficient of variation of the proportional error.

Each scenario was simulated and then estimated 200 times using a first-order conditional estimation with interaction method in NONMEM 7.4 (Icon Development Solutions) and SSE (stochastic simulation and estimation) of PSN (Perl-speaks-NONMEM, version 4.8.1, https://uupharmacometrics.github.io/PsN/). R (version 3.6.3) was used for preprocessing and visualization of the modeling results.

### 4.2. Bias and Precision of Parameter Estimates

The bias and precision of the parameter estimates in 72 scenarios were evaluated using rBias and rRMSE, respectively, to compare estimation performance.

$$\mathrm{rBias}=100\%\frac{1}{N}\underset{i}{\sum }\frac{P_{E}-\;P_{S}}{P_{S}}$$

$$\mathrm{rRMSE}=100\%\sqrt{\frac{1}{N}\underset{i}{\sum }\frac{{\left(P_{E}-\;P_{S}\right)}^{2}}{P_{S}^{2}}}$$

where PE is the fixed- or random-effect parameter estimate, and PS is the true fixed- and/or random-effect parameters (θ, ω2, θε) used for the PK profile simulation. The criteria for accuracy and precision were less than or equal to 15% and 35%, respectively [12,13,14].
